# Combined treatment with valsartan and fluvastatin to delay disease progression in nonpermanent atrial fibrillation with hypertension: A clinical trial

**DOI:** 10.1002/clc.23487

**Published:** 2020-10-26

**Authors:** Zhiqiang Zhao, Yu Yang, Jianwei Wang, Zhaojie Dong, Xiaowei Niu, Enzhao Liu, Tong Liu, Lifeng Li, Yingzi Liang, Guangping Li

**Affiliations:** ^1^ Tianjin Key Laboratory of Ionic‐Molecular Function of Cardiovascular disease, Department of Cardiology Tianjin Institute of Cardiology, Second Hospital of Tianjin Medical University Tianjin China; ^2^ Department of Respiratory and Critical Care Medicine Tangshan Gongren Hospital Tangshan Hebei China; ^3^ Department of Cardiology An Zhen Hospital affiliated to Capital Medical University Beijing China; ^4^ Department of Geriatrics the first hospital of Shijiazhuang City Hebei China

**Keywords:** 7‐day Holter monitoring, atrial fibrillation, fluvastatin, randomized controlled clinical trial, valsartan

## Abstract

**Background:**

Atrial fibrillation (AF) is a complex cardiac arrhythmia in clinical practice with increasing incidence. However, the effects of statins on patients with AF are not quite clear.

**Hypothesis:**

To investigate the protective effect of calcium channel blocker (CCB) and valsartan combined fluvastatin on hypertension (HTN) patients with nonpermanent AF.

**Methods:**

In three and a half years, 189 cases of patients diagnosed as HTN combining nonpermanent AF by eight medical centers, were recruited and randomly assigned to four groups with varied treatments: CCB group; CCB + statin group; valsartan group; and valsartan + statin group. The four groups were followed up for 24 months. The 7‐day Holter ultrasound echocardiography (UCG) and biochemical indexes were completed at preset time nodes respectively.

**Results:**

After 24 months of follow‐up, 178 patients completed the study. Compared with CCB group, the blood lipid level, inflammatory index, ultrasonic index and electrocardiographic measurement results of CCB + statin group, valsartan group and valsartan + statin group were improved in different degrees and had statistical significance (*P* < .05 or *P* < .01). Furthermore, the improvement trend of CCB + statin group and valsartan + statin group was more obvious.

**Conclusions:**

The results indicated that valsartan can reduce AF load and recurrence rate, and delay the progression of nonpermanent AF to permanent AF in multiple ways, and the effect of combination of valsartan and fluvastatin is more significant. These results provide a new direction for the integrated upstream control strategy of AF.

## 1 INTRODUCTION

Atrial fibrillation (AF) is a complex cardiac arrhythmia in clinical practice with increasing incidence.^1^ Thromboembolism, as a familiar but early‐onset complication of AF, significantly increases the disability and mortality of AF patients,^2^ and causes a heavy burden on their daily life and social economy.^3^, ^4^ Hypertension (HTN) is deemed to be an independent risk factor since its harmful effects like activating sympathetic nerve, increasing pressure of the left atrium and activating the renin‐angiotensin‐aldosterone system (RAAS), which further leading to autonomic nerve changes, cardiac fibrosis and atrial remodeling.^5^, ^6^ Due to AF cannot be completely cured by interventional surgery or common drugs, nowadays researchers are trying to find the upstream control strategy and preventive therapy for delaying the occurrence and development of AF.^7^ Statins are widely used as an optimal treatment for elderly AF patients because of their combined cardiovascular diseases like coronary heart disease (CHD), heart failure (HF), HTN and hyperlipidemia in clinical practice. An earlier animal experiment suggests the potential therapeutic effect of statins on AF by developing inflammatory response and oxidative stress.^8^ However, the effects of statins on patients with AF are not quite clear. In this study, four groups with varied treatments were designed to investigate protective effects of antihypertensive drugs and statins on HTN patients with nonpermanent AF.

## 2 PARTICIPANTS AND METHOD

### 2.1 Ethical principles and registration

Our study follows the declaration of Helsinki. We obtained the written informed consent from each patient before the trial. Before performance of this trial, the study protocol, informed consent of patients, case report form and other essential materials have been submitted to the ethics committee of the Second Hospital of Tianjin Medical University (IEC). The study has been reviewed and approved by IEC (Clinical Review, 2012, No.27) and has been registered in the Chinese Clinical Trial Registry (registration number: ChiCTR‐TRC‐12002642). The article for relevant trial scheme has been published.^9^

### 2.2 Inclusion and exclusion criteria

We recruited 189 patients who have been diagnosed as HTN combined with nonpermanent AF by outpatient or inpatient department from eight medical centers in China between February 2013 to July 2016. The eight medical centers are listed as follows: The Second Hospital of Tianjin Medical University, People's Hospital of Nankai University, Tianjin Dongli Hospital, Tianjin Jinghai Hospital, Tianjin Beichen Hospital of Tradition Chinese Medicine, Tianjin Port Hospital, First Hospital of Qinhuangdao and Tangshan Gongren Hospital.

Entry criteria were (1) HTN, defined as the systolic blood pressure (SBP) ≥ 140 mmHg (1 mmHg = 0.133kpa) and/or diastolic blood pressure (DBP) ≥ 90 mmHg continuously measured twice in a s resting state, or the patient who has blood pressure controlled as less than 180/110 mmHg by using antihypertensive drugs since his/her clear history of HTN; (2) at least two symptomatic AF attacks recorded by electrocardiogram (ECG) within 6 months before enrollment, and the duration of AF was less than 1 year. In addition, at the time of enrollment, abnormal AF rhythm could be converted to normal sinus rhythm independently or by medical intervention. However, drug and/or electrical cardioversion must be performed between 6 months and 8 weeks before enrollment, and no cardioversion was undertaken in the last 8 weeks; (3) did not take angiotensin converting enzyme inhibitors (ACEIs), angiotensin receptor blockers (ARBs) and/or statins for more than 2 weeks before enrollment, or taking the above drugs, but can withstand the washout period of more than 2 weeks after discontinuation; (4) aged between 25 to 79 years; and (5) signing informed consent voluntarily.

Exclusion criteria were (1) permanent AF or persistent AF lasting for more than 1 year; (2) unstable angina pectoris (UAP) with poor controlled; (3) severe left main coronary artery disease confirmed by coronary angiography (CAG); (4) acute myocardial infarction (AMI) within 3 months; (5) grade III or IV HF (NYHA heart function grade); (6) valvular disease with indications of surgical operation or interventional therapy; (7) stroke or transient ischemic attack (TIA) within 3 months; (8) thyroid disease with uncontrollable complications (abnormal FT3, FT4 or TSH, or essential thyroid treatment taken during the time of enrollment); (9) severe liver or renal function damage defined as alanine aminotransferase (ALT) more than 80 U/L, aspartate aminotransferase (AST) more than 80 U/L or creatinine (Cr) more than 1.5 mg/dL; (10) severe hyperlipidemia needed essential treatment with statins/bates defined as total cholesterol (TC) more than 6 mmol/L or low density lipoprotein cholesterol (LDL‐C) more than 3.36 mmol/L; (11) Other ARB, ACEI and/or statins have been taken and cannot be washed for more than 2 weeks; (12) contraindications for statins and/or ARBs; (13) pregnant/planned pregnant women or lactating women; (14) the possibility of poor compliance or noncooperation during holistic follow‐up; and (15) inappropriate age.

### 2.3 Group design and therapy schedule

Participants were randomly assigned to be treated either with dihydropyridine CCB (CCB group, n = 45), CCB combined with fluvastatin (CCB + statin group, n = 48), valsartan (valsartan group, n = 46), or valsartan combined with fluvastatin (valsartan + statin group, n = 50). Patients' medical history, medication history (including drug type, daily dose, starting time and ending time of medication) and results of physical examination were recorded before drug intervention. Furthermore, resting ECG, 7‐day electrocardiogram (7‐day Holter), UCG, related blood routine, whole blood biochemistry and other laboratory examinations were also tested. In this study, valsartan (Diovon, Beijing Novartis Pharmaceutical Co., Ltd.) was recommended to take in the morning every day as an initial dose of 80 mg; fluvastatin (Lescol, Beijing Novartis Pharmaceutical Co., Ltd.) had an initial dose of 40 mg, once a day, before going to bed; and dihydropyridine CCB was only allowed to use amlodipine, nifedipine or felodipine. After patients entering the group, the dosage of each group could be adjusted according to the clinical situation. However, all the patients must meet the standard of blood pressure control as 140/90 mmHg. The recommended dosage of valsartan and fluvastatin were ≥ 80 mg and ≥ 40 mg, respectively.

### 2.4 Data collection and follow‐up

Subjects filled in their case report form at the beginning of the trial, after that their gender, age, body mass index (BMI, = weight/height2, kg/m2), history of diabetes, stroke, smoking or drinking, UCG, resting ECG, 7‐day Holter as well as blood routine, blood lipid, myocardial enzyme, liver and renal function and other blood biochemical laboratory test results were recorded in detail. The combined medication and adverse events were observed within 24 months of follow‐up. During this period, all the selected patients were followed up once every 3 months to review blood pressure, ECG, and cardiac function classification (NYHA). In addition, patient logs, disease changes, new combined drugs, adverse events and doctor's recommended prescriptions were also recorded. At the 24th month, UCG, related blood routine test, whole blood biochemistry and other laboratory tests were reexamined for comparison. Holter was reexamined at the 6th, 12th and 24th months. For the 7‐day Holter, 22:00‐06:00 am AF attack time was selected to measure the f‐f interval. In detail, successive 20 f‐f intervals that from the second or the third f wave after either QRS wave were measured in order to avoid the influence of QRS complex wave, T wave and U wave. Furthermore, in the same period, lead III was selected to measure the amplitude of wave. Similarly, consecutive 20 f waves were recorded. After that the average of these measured value was taken for comparison. It is worth noting that the f‐f interval refers to the distance between the starting point of the previous f wave and the starting point of the next f wave while f wave amplitude means the distance from the upper edge of f‐wave peak to the upper edge of the valley. At last, AF load was defined as the total time of AF attack recorded in 7‐day Holter as a percentage of the total recording time.

### 2.5 Statistical methods

SPSS 20.0 was utilized to analyze the data. The measuring data of near normal distribution were described by mean standard ± deviation and the counting data was expressed by percentage. The comparison of changes in before and after treatments in each group was made by Student's *t* test and multiple comparisons of groups were made by one‐way ANOVA followed by Bonferroni correction. *P* values below .05 were considered significant.

## 3 RESULT

Eleven participants quit the study during the observation and treatment. In CCB group, there were six patients withdrew from the trial because of intolerable edema after taking CCB drugs (n = 2), prolonged QT interval more than 0.5 s (n = 2) or severe sinus bradycardia (n = 2). Only one patient withdrew since liver dysfunction in CCB + statin group. In valsartan group, two cases quit due to poor blood pressure control that needed to use other antihypertensive drugs except ARB (n = 1) or abnormal renal function (n = 1). In valsartan + statin group, one patient quit because of AMI while the other patient withdrew since severe sinus bradycardia. After the above patients were excluded, 41, 45, 44, and 48 patients in CCB group, CCB + statin group, valsartan group and valsartan + statin group were available for analysis. According to the rules of Statistics, the proportion of exfoliated cases was acceptable so that it would not make a significant negative impact on the research results.

### 3.1 General clinical data

Finally, a total of 178 HTN patients with nonpermanent AF were followed up, including 94 males and 84 females, aged 44 to 77 years (mean 67.06 ± 8.28). There were 28 patients with diabetes, 23 patients with stroke, 74 patients with smoking history, and 51 patients with drinking history. There was no significant difference in age, gender, BMI, smoking or drinking history, diabetes, stroke and lung disease history among the four groups (*P* > .05). No significant difference was found in mean SBP and DBP among the four groups (*P* > .05). In addition, at the end of follow‐up, the blood pressure of all the four groups was lower than that before treatment. However, the difference was not statistically significant, and there was no significant difference between the groups (*P* > .05).

### 3.2 Drug applications

After adjustment of medication by follow‐up doctors, no significant difference was observed in the use of antiplatelet drugs, anticoagulant drugs and nitrates drugs in each group during the follow‐up period (*P* > .05, Table 1).

**TABLE 1:** Comparison of medication between four groups (n, %)

Medications	CCB	CCB + statin	Valsartan	Valsartan + statin	*P* value
Antiplatelet drugs	22 (53.66)	23(51.11)	18 (40.91)	23 (47.92)	.358
Anticoagulant drugs	25 (60.98)	24(53.33)	22 (50.00)	26 (54.17)	.362
Nitrates	10 (24.39)	11(24.44)	7 (15.91)	10 (20.83)	.436
β‐blocker	15 (36.59)	12(26.67)	10 (22.73)	13 (27.08)	.185
Amiodarone	26 (63.41)	26(57.78)	29 (65.91)	34 (70.83)	.613
Propafenone	12 (29.27)	10(22.22)	10 (22.73)	12 (25.00)	.535
Antiarrhythmic TCM	10 (24.39)	11(24.44)	10 (22.73)	13 (27.08)	.916

*Note*: Data were expressed as percentage of medications in each group. TCM, traditional Chinese medicine.

### 3.3 Biochemistry and UCG

After 24 months treatment, the levels of serum TC, triglyceride (TG) and LDL‐C in CCB + statin group and valsartan + statin group were obviously decreased and were significantly lower than those in CCB group and valsartan group (*P* < .05). At the beginning of enrollment, there was no significant difference in the inflammatory index (high‐sensitivity C‐reactive protein, hs‐CRP), liver and renal function, myocardial enzyme, N‐terminal brain natriuretic peptide (NT‐proBNP) or blood lipid level among the groups (*P* > .05). After 24 months of treatment, the levels of hs‐CRP (mg/L) in CCB + statin group, valsartan group and valsartan + statin group were lower than those before treatment (3.40 ± 1.05 vs 6.85 ± 1.27, 3.39 ± 1.06 vs 6.76 ± 1.31, 3.29 ± 1.02 vs 6.94 ± 1.03, respectively; all *P* < .01), and were significantly lower than that in CCB group (*P* < .05). However, no significant difference was noted in hs‐CRP between CCB + statin group, valsartan group and valsartan + statin group (*P* > .05). In addition, the level of NT‐proBNP (ng/L) in valsartan + statin group was significantly decreased after combined treatment (315.44 ± 50.45 vs 290.35 ± 36.13; *P* < .05). There were no significant changes in liver or renal function and myocardial enzyme level in each group compared with those before treatment, and no significant difference was observed between the groups (*P* < .05, Table 2).

**TABLE 2:** Comparison of biochemistry and UCG results of four groups ($\bar{x}\pm s$)

Variable	CCB (n = 41)			CCB + statin (n = 45)			Valsartan (n = 44)			Valsartan+statin (n = 48)	
	Before	After		Before	After		Before	After		Before	After
Cr (umol/L)	72.27 ± 19.51	72.05 ± 16.20		72.69 ± 15.64	72.86 ± 14.56		72.93 ± 15.40	72.91 ± 13.04		71.89 ± 14.49	72.47 ± 12.06
ALT (U/L)	21.30 ± 8.33	21.20 ± 7.19		21.47 ± 9.30	21.06 ± 6.99		21.46 ± 9.33	21.14 ± 7.07		21.81 ± 8.01	21.11 ± 6.54
TC (mmol/L)	5.10 ± 0.46	5.24 ± 0.33		5.13 ± 0.50	4.22 ± 0.47^a^ ^,^ ^c^		5.20 ± 0.54	5.18 ± 0.34		5.19 ± 0.53	4.20 ± 0.42^a^ ^,^ ^c^
TG (mmol/L)	1.80 ± 0.29	1.84 ± 0.36		1.83 ± 0.31	1.41 ± 0.20^a^ ^,^ ^c^		1.83 ± 0.27	1.81 ± 0.37		1.82 ± 0.27	1.43 ± 0.19^a^ ^,^ ^c^
LDL‐C (mmol/L)	3.05 ± 0.27	3.00 ± 0.25		2.99 ± 0.27	2.45 ± 0.24^a^ ^,^ ^c^		3.06 ± 0.31	2.98 ± 0.35		3.02 ± 0.32	2.48 ± 0.29^a^ ^,^ ^c^
hs‐CRP (mg/L)	6.89 ± 1.35	6.59 ± 0.95		6.85 ± 1.27	3.40 ± 1.05^a^ ^,^ ^c^		6.76 ± 1.31	3.39 ± 1.06^a^ ^,^ ^c^		6.94 ± 1.03	3.29 ± 1.02^a^ ^,^ ^c^
CK‐MB (IU/L)	13.20 ± 4.75	13.27 ± 4.78		13.25 ± 4.27	13.67 ± 4.52		13.51 ± 4.11	13.46 ± 4.02		13.53 ± 3.93	13.29 ± 3.79
NT‐ProBNP (ng/L)	296.37 ± 36.88	300.49 ± 38.75		301.02 ± 58.62	305.24 ± 53.56		306.41 ± 46.93	287.48 ± 38.11		315.44 ± 50.45	290.35 ± 36.13^b^
IVST (mm)	9.54 ± 2.02	10.69 ± 3.17		9.67 ± 2.53	9.52 ± 2.16		9.19 ± 1.88	8.15 ± 1.12^c^		11.03 ± 1.98	7.69 ± 2.07^b^ ^,^ ^c^
LVEDD (mm)	46.52 ± 3.92	47.72 ± 2.89		45.75 ± 3.23	44.43 ± 2.56		46.72 ± 5.45	43.62 ± 5.26		50.01 ± 6.89	39.42 ± 9.22^b^
LVPW (mm)	9.37 ± 1.62	9.56 ± 1.51		9.28 ± 1.78	9.15 ± 1,81		8.77 ± 1.66	8.69 ± 1.05		9.07 ± 1.85	8.17 ± 2.34
LAD (mm)	37.27 ± 2.72	36.83 ± 1.89		37.42 ± 2.81	32.72 ± 3.05^a^ ^,^ ^c^		36.68 ± 6.84	34.76 ± 4.72^b^ ^,^ ^c^		37.91 ± 3.13	32.26 ± 2.23^b^ ^,^ ^c^
LVEF (%)	48.45 ± 5.92	48.65 ± 2.86		49.67 ± 4.71	50.18 ± 5.12		47.46 ± 3.26	48.72 ± 4.39		48.83 ± 5.49	50.10 ± 5.37

Abbreviations: ALT, alanine aminotransferase; Cr, creatinine; CK‐MB, creatine kinase isoenzyme; IVST, interventricular septal thickness; LDL‐C, low density lipoprotein cholesterol; LVEDd, left ventricular end‐diastolic diameter; LVPW, left posterior wall; LAD, left atrial diameter; LVEF, left ventricular ejection fraction; TC, total cholesterol; TG, triglyceride; hs‐CRP, high sensitivity C‐reactive protein; NT‐proBNP, N‐terminal brain natriuretic peptide.

*Note*: Data were expressed as mean ± SD.

^a^ *P* < .01 compared with before treatment.

^b^ *P* < .05 compared with before treatment.

^c^ *P* < .05 compared with group A after treatment.

Before the treatment, no significant difference in interventricular septum thicknessivst (IVST), left ventricular end‐diastolic diameter (LVEDd), left ventricular posterior wall (LVPW), left atrial diameter (LAD) or left ventricular ejection fraction (LVEF) was found among the groups (*P* > .05). But after 24 months of treatment, the measured values of LAD in CCB + statin group, valsartan group and valsartan + statin group were obviously decreased (*P* < .05 or *P* < .01). Unlike CCB group, the values of LAD and IVST in valsartan group and valsartan + statin group were significantly reduced after medication (*P* < .05). It is worth mentioning that the values of IVST, LVEDd and LAD in valsartan + statin group were significantly decreased after 24 months combined intervention (7.69 ± 2.07 vs 11.03 ± 1.98, 39.42 ± 9.22 vs 50.01 ± 6.89, 32.26 ± 2.23 vs 37.91 ± 3.13, respectively; all *P* < .05). Although LVEF of each group was higher than before, no significant difference was observed (*P* > .05, Table 2).

### 3.4 f‐f interval and f wave amplitude

In all the four groups, 7‐day holter illustrated that there was no significant difference in the f‐f interval or f wave amplitude at the beginning of the trial (*P* > .05) while reexamined 7‐day holter demonstrated the f‐f interval was gradually shortened and f wave amplitude was gradually increased at 6, 12, and 24 months after corresponding treatment. In detail, reexamined holter indicated that the f‐f intervals of CCB + statin group, valsartan group and valsartan + statin group were significantly longer than that of CCB group (*P* < .05) while no significant difference between CCB + statin group and valsartan + statin group was noted (*P* > .05). However, there was no noticeable change of the f‐f interval in CCB group after drugs intervention (*P* > .05). Similarly, the amplitudes of f‐wave in CCB + statin group, valsartan group and valsartan + statin group were significantly higher after 24 months treatment (*P* < .05) while no significant difference was found in CCB group (*P* > .05). In addition, in CCB group, the f‐f interval moderately shortened while f wave amplitude gradually decreased during the follow‐up period. Nevertheless, in valsartan group and valsartan + statin group, their f‐f intervals extended gradually while their amplitudes of f‐wave increased slightly in the same period. Furthermore, the amplitudes of f‐wave and f‐f intervals in valsartan group and valsartan + statin group were significantly higher and longer than those in CCB group after 24 months of treatment (*P* < .05). Interestingly, holter suggested the f‐f interval of the 12th month in CCB group was significantly shorter than those before treatment, 6 or 24 months after treatment, therefore the specific reasons for this abnormal phenomenon need to be further studied by expanding the sample (Table 3).

**TABLE 3:** Comparison of f‐f interval and f wave amplitude in four groups ($\bar{x}\pm s$)

Groups	f‐f interval (ms)					f wave amplitude (mV)			
	Before	6 months	12 months	24 months		Before	6 months	12 months	24 months
CCB	140.89 ± 24.97	138.43 ± 18.87	122.27 ± 17.91^b^	133.35 ± 19.62		0.095 ± 0.013^a^	0.091 ± 0.016	0.088 ± 0.012	0.089 ± 0.011
CCB + statin	134.54 ± 12.18	132.62 ± 23.65	155.43 ± 15.51^b^ ^,^ ^c^	162.59 ± 18.49^b^ ^,^ ^c^		0.091 ± 0.019	0.093 ± 0.017	0.103 ± 0.019^b^ ^,^ ^c^	0.111 ± 0.021^b^ ^,^ ^c^
Valsartan	136.45 ± 21.83	137.97 ± 17.13	152.23 ± 13.37^b^ ^,^ ^c^	163.51 ± 17.65^b^ ^,^ ^c^		0.087 ± 0.018	0.094 ± 0.013	0.103 ± 0.013^b^ ^,^ ^c^	0.105 ± 0.012^b^ ^,^ ^c^
Valsartan+statin	138.37 ± 19.79	139.69 ± 16.55	151.17 ± 18.13^b^ ^,^ ^c^	164.05 ± 16.43^b^ ^,^ ^c^		0.091 ± 0.015	0.097 ± 0.015	0.107 ± 0.014^b^ ^,^ ^c^	0.109 ± 0.013^b^ ^,^ ^c^

*Note*: Data are expressed as mean ± SD.

^a^ *P* < .01 compared with before treatment.

^b^ *P* < .05 compared with before treatment.

^c^ *P* < .05 compared with group A after treatment.

### 3.5 AF load and mean heart rate

After 24 months of grouping treatment, the AF loads of the four groups were higher than those before medication, but the levels of AF load in CCB + statin group, valsartan group and valsartan + statin group were significantly lower than that in CCB group (55.26 ± 42.13 vs 66.87 ± 39.35, 54.88 ± 47.69 vs 66.87 ± 39.35, 45.74 ± 46.86 vs 66.87 ± 39.35, respectively; all *P* < .01). Similarly, the mean heart rates of the four groups decreased after treatment while the trends of decline were more obvious in valsartan group and valsartan + statin group as significant decreased at the end of follow‐up (62.35 ± 3.91 vs 72.93 ± 4.13, 61.18 ± 4.33 vs 72.93 ± 4.13, respectively; all *P* < .05, Table 4).

**TABLE 4:** Comparison of AF burden and mean HR in four groups ($\bar{x}\pm s$)

Items	CCB (n = 41)			CCB + statin (n = 45)			Valsartan (n = 44)			Valsartan + statin (n = 48)	
	Before	After		Before	After		Before	After		Before	After
AF dur%	23.88 ± 16.93	66.87 ± 39.35^a^		24.12 ± 13.25	55.26 ± 42.13^a^		21.89 ± 11.69^b^	54.88 ± 47.69^a^ ^,^ ^c^		22.43 ± 10.69	45.74 ± 46.86^a^ ^,^ ^c^
HR	76.09 ± 5.55	72.93 ± 4.13		79.15 ± 5.26	65.82 ± 5.03^a^		78.94 ± 6.15	62.35 ± 3.91^a^ ^,^ ^c^		77.28 ± 6.15	61.18 ± 4.33^a^ ^,^ ^c^

Abbreviations: AF, atrial fibrillation; CCB, calcium channel blocker.

*Note*: Data are expressed as mean ± SD; AF dur%, AF burden (%); HR, heart rate.

^a^ *P* < .01 compared with before treatment.

^b^ *P* < .05 compared with before treatment.

^c^ *P* < .05 compared with group A after treatment.

## 4 DISCUSSION

Cardiogenic thrombus caused by atrial fibrillation (AF) accounts for about one third of the causes of ischemic stroke. Stroke may even attack before the patient is diagnosed with AF.^10^ The high incidence of thromboembolism in AF significantly increased the morbidity and mortality of patients and further increased the economic burden and affected the quality of life.^11^ Therefore, it has important clinical significance to strengthen the control of AF risk factors and delay the pathologic progression of paroxysmal AF to persistent or permanent AF with the social structure of population aging worldwide. The conventional clinical risk factors for increased incidence rate of AF include age, valvular heart disease, HTN, HF, obesity, diabetes, alcohol consumption, etc.^12^ HTN, especially excessive SBP, is an independent risk factor for AF,^13^, ^14^ while RAAS may be closely related to the occurrence and development of AF in patients with HTN.^15^ In addition, our previous studies also confirmed that inhibition of RAAS is beneficial to prevent AF attack, suggesting that RAAS may play an important role in AF atrial remodeling.^16^, ^17^

To block RAAS by ARBs has a beneficial effect in reducing the incidence of AF.^18^ A cohort study by Marott et al.^19^ demonstrated that compared with ACEI, ARB had a better effect on the prevention of new‐onset AF in HTN with AF. In addition, Hsieh et al.^20^ also found that the above impacts of ARB were better than those of ACEI in patients with HTN and multiple AF risk factors (including congestive HF, diabetes, valvular heart disease, etc), especially in patients with previous history of stroke or TIA. Valsartan, a representative ARB drug, can selectively block the binding of AngII and AT1 receptor, block RAAS, improve left ventricular hemodynamics, alleviate atrial enlargement, directly regulate ion channels, and reduce the electrical remodeling of damaged cardiac myocytes. Results of VALUE study suggested that compared with amlodipine based treatment, valsartan can decrease the incidence of new‐onset AF, especially the incidence of persistent AF in patients with HTN.^21^ Cui et al.^22^ treated 120 AF patients with placebo, 80 or 160 mg/d valsartan respectively after radiofrequency ablation and the results showed that valsartan could significantly reduce the risk of AF recurrence in a dose‐dependent manner. In our study, the measured values of LAD and levels of hs‐CRP in subjects that treated with valsartan were significantly lower than those treated with CCB (*P* < .05). In addition, after 24 months of treatment, the f‐f interval and f wave amplitude in valsartan group were longer and higher than those in the group that treated with CCB only. These results further confirmed that long‐term application of valsartan could effectively delay or even reverse left atrial enlargement and reduce atrial remodeling, improve left ventricular function, reduce sympathetic tension, enhance vagus activity, inhibit inflammation and thus reduce the incidence of AF in HTN patients.

Statins, as secondary prevention drugs for coronary heart disease, can not only significantly reduce blood lipid, but also effectively decrease the incidence and mortality of cardiovascular events by regulating serum lipid levels and stabilizing artery plaque.^23^ In recent years, adding statins as the optimal treatment has been proved to be beneficial to AF.^24^ Statins can significantly improve the prognosis of patients with AF and independently reduce the risk of all‐cause death.^25^ Hung et al.^26^ observed that the statins can significantly reduce the risk of new‐onset AF in elderly HTN patients those aged ≥64 years, and the patients with CHADS2 score ≥ 2 benefited more than those with a score of 1. In addition, Ma et al.^27^ confirmed that statins can reduce the risk of AF in the elderly aged ≥65 years by about 19%. Furthermore, Kunt et al.^28^ showed that atorvastatin can significantly reduce the incidence of AF and the mortality of cardiovascular events in those elderly patients who had underwent coronary artery bypass grafting (CABG) before. All the above researches suggested that statins can play an antiarrhythmic effect that independent from regulating blood lipids. Similarly, our results showed that compared with CCB group, the level of hs‐CRP in CCB + statin group was significantly decreased after treatment with fluvastatin (*P* < .01); the level of hs‐CRP in valsartan group after treatment with valsartan was also decreased, but the trend in valsartan + statin group with fluvastatin was more obvious (Table 2). The antiarrhythmic function of statins is deemed to be closely related to its beneficial effect on anti‐inflammatory and anti‐oxidative stress response, reducing active oxygen radical production and improving atrial remodeling caused by the rise of AngII, so as to reduce the recurrence and to delay the progression of AF.^29^ Our previous study has also shown that fluvastatin is beneficial for maintaining normal sinus rhythm in patients with nonpermanent AF.^30^ Furthermore, Inohara et al.^31^ suggested that the progression and recurrence of AF are closely related to BNP level. In this study, we showed that compared with using valsartan alone, the combination of valsartan and fluvastatin can improve the heart structure as a significant decrease in NT‐proBNP (*P* < .05). The combined treatment also improved inflammatory response and maintained heart rate variability with normal sinus rhythm. These results indirectly confirmed the role of statins in the prevention and treatment on AF.

It is worth noting that in this study, we innovatively investigated the different effects of four groups drug interventions on the f‐f interval and amplitude of f‐wave. The shortening of the f‐f interval that recorded by ECG can be used as an effective index to predict the prolonged duration of paroxysmal AF and to reflect the electrical remodeling of atrium while the down regulated changes in amplitudes of f‐wave may be related to the recurrence of AF. It is apparent to find in valsartan + statin group, the trends of prolongation in f‐f interval and increase of f wave amplitude were more obvious. Compared with heart rate, which is a common index, electrocardiographic indexes f‐f interval and amplitude of f‐wave can interpret the beneficial effect of combined application of valsartan and fluvastatin on HTN patients with nonpermanent AF in a deeper level.

**FIGURE 1:**
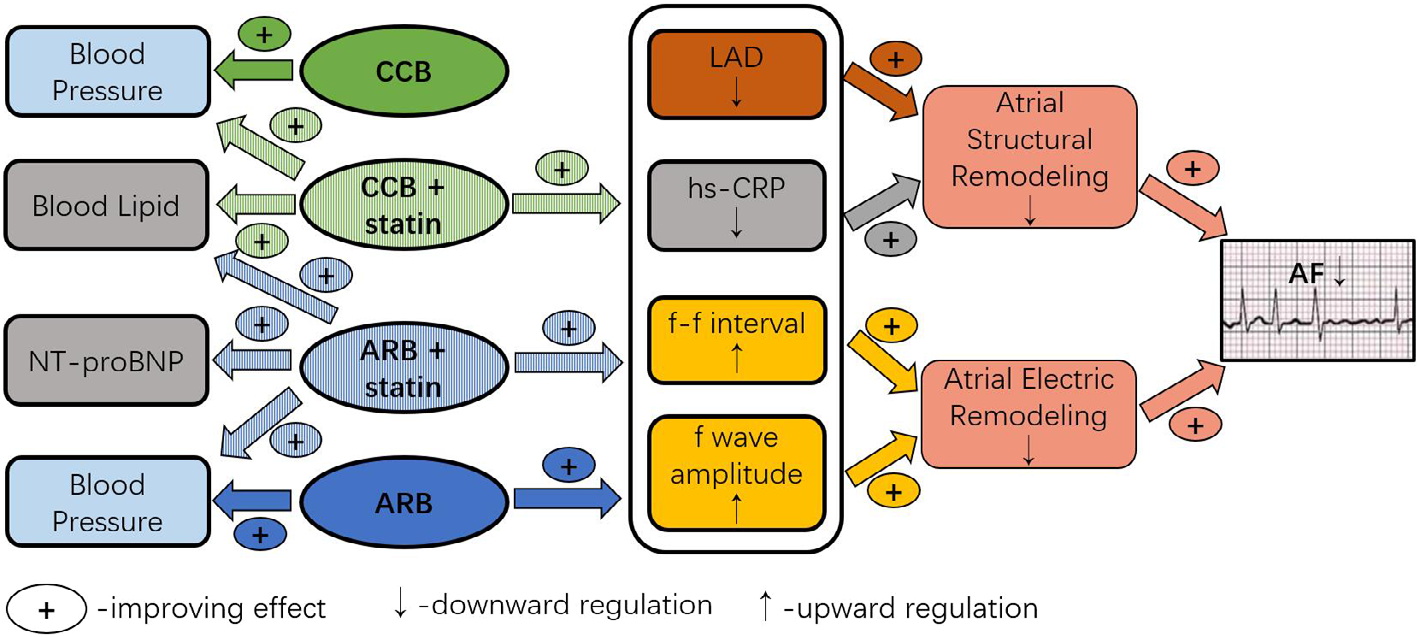
Comparison of four groups of drug mechanism

As mentioned above, existing interventions cannot completely cure AF, and paroxysmal AF always progresses to permanent AF. Our results were consistent with the above consensus. After 24 months of follow‐up, the heart rate of the four groups decreased, but the AF load increased significantly. However, the AF load in valsartan group and valsartan + statin group was significantly lower than that in CCB group after treatment (all *P* < .01, Table 4), which indicated that using valsartan alone or combined with fluvastatin could prolong the time from nonpersistent AF to persistent AF. It is worth mentioning that in our results of echocardiography, the measured values of left atrial diameter (LAD) of CCB + statin group, valsartan group and valsartan + statin group were significantly reduced, but the measured values of thickness interventricular septal thickness (IVST) and left ventricular end‐diastolic diameter (LVEDd) of valsartan + statin group were improved additionally (all *P* < .05, Table 2). These results suggest that the protective effect of valsartan combined with fluvastatin on AF load may be achieved by improving atrial remodeling (Figure 1).

There are also some limitations in this study. First, although this study is a multicenter clinical study, there are few selected cases, and the follow‐up time is short, which affects the persuasion of the test results. Further larger randomized clinical trial will be necessary to confirm the conclusions of our study. Secondly, this study did not assess the effect of application of fluvastatin alone on AF, and failed to directly prove whether fluvastatin can prevent AF independently. At last, although 7‐day Holter can capture most AF episodes, the potential subclinical AF may be underestimated.

To sum up, our study is a multicenter, randomized, open, four group parallel design clinical trial to investigate whether valsartan and its combination with fluvastatin can reduce the recurrence of paroxysmal AF in patients with HTN and nonpermanent AF and delay the progression of nonpermanent AF to permanent AF compared with CCB or CCB combined with fluvastatin. Our results suggest that valsartan can decrease the occurrence of AF by reducing myocardial load, inhibiting atrial remodeling, exerting anti‐inflammation, and regulating nerve tension. Similarly, fluvastatin has the effects of anti‐inflammatory and improving atrial remodeling. The combination of valsartan and fluvastatin can significantly improve heart function, reduce the AF load, maintain normal sinus rhythm, and delay the time of nonpermanent AF to permanent AF. It is obvious that the combination of the two drugs can produce synergistic therapeutic impact and improve the therapeutic effect. RAAS blocker combined with statins is a highlight of nontraditional antiarrhythmic drugs in the treatment of cardiac arrhythmia. With further validation of basic research and large‐scale clinical trial, RAAS blocker combined with statins may play a certain role in the prevention and treatment of AF, which is expected to become a new strategy for AF upstream control.

## CONFLICT OF INTEREST

The authors declare that there is no conflict of interest.

## DATA AVAILABILITY

The data used to support the findings of this study are available from the corresponding author upon request.
